# Acute therapy-related myelodysplastic syndromes following capecitabine and oxaliplatin therapy in gastric malignant tumor: A case report

**DOI:** 10.1097/MD.0000000000039049

**Published:** 2024-07-26

**Authors:** Yi-fan Qian, Hang-ping Chen, Guo-fei Ren

**Affiliations:** a Department of Pharmacy, Sir Run Run Shaw Hospital, School of Medicine, Zhejiang University, Hangzhou, Zhejiang Province, China; b Department of Pharmacy, Affiliated Xiaoshan Hospital, Hangzhou Normal University, Hangzhou, China.

**Keywords:** capecitabine, gastric cancer, myelodysplastic syndrome, oxaliplatin

## Abstract

**Rational:**

Patients with gastric cancer show a relatively low incidence of developing secondary myelodysplastic syndrome (MDS).

**Patient concerns:**

A 60-year-old man was admitted because of pain and discomfort in the upper abdomen and intermittent abdominal pain.

**Diagnoses:**

Ulcerative moderately poorly differentiated adenocarcinoma (pT2N2M0G3, stage IIB) and MDS.

**Interventions:**

The patient underwent chemotherapy with oxaliplatin (OXP, intravenously guttae on day 1) plus capecitabine (CAP, bis in die orally on day 1–14). The patient developed degree III myelosuppression after OXP plus CAP chemotherapy and MDS was subsequently confirmed by diagnosis of the bone marrow biopsy. Temporary but significant hematological improvements were observed after the patient received corresponding treatment, which helped achieve remission and improve pancytopenia.

**Outcomes:**

The patient presented partial remission after corresponding treatment and no other complications have been recorded.

**Lessons:**

Acute MDS is an unusual adverse effect induced by OXP plus CAP chemotherapy. It is urgent to suggest implementing a supplementary assessment or examination for patients receiving these therapies in future cases.

## 1. Introduction

The XELOX regimen (capecitabine plus oxaliplatin) has showed significant survival advantages in gastric cancer (GC) patients with adverse effects like gastrointestinal toxicity, vomiting and diarrhea.^[1]^ The myelodysplastic syndromes (MDS) are characterized by clonal disorders with dysplasia of at least 1 myeloid cell line and indolent disease courses. While it is extremely rare that acute MDS develops during XELOX treatment. Here, we present a rare case of acute MDS in a GC patient treated with XELOX.

## 2. Case report

### 2.1. Case presentation

The male patient (60 years old) consulted in our hospital on February 2, 2021 with pain and discomfort in the upper abdomen and intermittent abdominal pain lasting for more than 8 months.

On February 5, 2021, the patient underwent laparoscope-assisted radical gastrectomy for gastric cancer. Combined with immunohistochemistry results, postoperative pathological records noted a finding of “ulcerative moderately poorly differentiated adenocarcinoma (pT2N2M0G3, stage IIB).” And the gene detection results showed the presence of AT-rich interaction domain 1A (ARID1A) and Tumor Protein P53 (TP53) mutations; findings consistent with phenotypic characteristics of myeloid precursor cells.

The patient then underwent 2 courses of chemotherapy with oxaliplatin (OXP, 240 mg for intravenously guttae on day 1) plus capecitabine (CAP, 2000 mg Q.M. and 1500 mg Q.N. orally on day 1–14) from March 12, 2021. The patient developed degree III myelosuppression after treatment, and laboratory analysis indicated a white blood cell count (WBC) of 9.36 × 10^9^/L and a platelet count (PLT) of 34 × 10^9^/L (Table 1). It was improved after platelet raising treatment with subcutaneous injection of recombinant human thrombopoietin at a daily frequency of once a day. Then treatment was changed to an adjusted therapy of 230 mg dosage OXP plus a twice-daily regimen of 1500 mg oral CAP tablets for another 1 course, during which patient developed degree III myelosuppression again. Subsequently, significant improvements were observed after receiving platelet raising treatment. During the 4th and 5th course of chemotherapy, the treatment regimen was adjusted to include 200 mg dosage OXP plus a twice-daily regimen of 1500 mg oral CAP tablets. Following each chemotherapy session, there was a noticeable decrease in platelet count, with the lowest point recorded at 22 × 10^9^/L. However, the patient’s platelet count improved significantly after receiving appropriate treatment.

**Table 1:** Laboratory data at the first time of therapy-related MDS.

Project	Amount
White blood cell (×10^9^/L)	9.36
Hemoglobin (g/L)	108
Platelets (×10^9^/L)	34

On July 26, the patient received the 6th course of chemotherapy consisting of 200 mg dosage OXP plus a twice-daily regimen of 1500 mg oral CAP tablets. Pre-chemotherapy laboratory analysis on July 23 indicated a WBC of 2.87 × 10^9^/L, hemoglobin (HGB) level of 111 g/L, and PLT of 152 × 10^9^/L. Following treatment with recombinant human granulocyte colony-stimulating factor, chemotherapy was initiated. Subsequent complete blood count (CBC) on July 30 revealed a WBC of 2.99 × 10^9^/L, HGB of 107 g/L, and a significant decrease in platelet count to 64 × 10^9^/L. CAP was temporarily discontinued, and a daily dose of 24 million units of recombinant human interleukin-11 injection was administered to elevate platelet levels, along with recombinant human granulocyte colony-stimulating factor injection to boost leukocyte counts. On August 2, treatment shifted to recombinant human thrombopoietin injection at a dosage of 15,000 units per day to elevate the platelet count. CBC reassessment on August 12 showed a WBC of 2.81 × 10^9^/L, HGB of 93 g/L, and PLT of 69 × 10^9^/L, indicating acceptable blood parameters. Therefore, the patient was discharged continuing to take CAP till the end of the course on August 22. However, the patient was readmitted on September 14, and CBC on admission revealed a WBC of 2.76 × 10^9^/L, HGB of 103 g/L, and a severely suppressed PLT of 11 × 10^9^/L (Grade IV), while there were no visible bruises or significant bleeding symptoms under physical examination. Immediate administration of recombinant human thrombopoietin injection at a dosage of 15,000 units was initiated, along with tranexamic acid infusion injection (intravenously guttae) at a dosage of 1g per day to prevent bleeding. Subsequent CBC reassessment on September 22 showed a WBC of 2.63 × 10^9^/L, neutrophils of 1.56 × 10^9^/L, HGB of 92 g/L, and PLT of 21 × 10^9^/L. Treatment to elevate leukocyte and PLT was continued. Platelet antibody testing on September 24 revealed positive antiplatelet antibodies, with IgA levels at 4.1 ng/ml, IgG levels at 21.8 ng/ml, and IgM levels at 5.4 ng/ml. On September 25, CBC reassessment indicated a WBC of 2.08 × 10^9^/L, neutrophils of 0.98 × 10^9^/L, HGB of 91 g/L, and PLT of 14 × 10^9^/L. Physical examination on September 28 revealed small bleeding spots on the patient’s lower extremities, and the result of hematological consultation revealed that post-chemotherapy bone marrow suppression was considered, leading to further bone marrow examination. Platelet transfusion and elevation treatment were continued, with 16 units of platelets transfused on September 28. CBC reassessment on September 30 indicated a slight improvement in PLT. On October 1, bone marrow examination revealed active hematopoiesis with rare megakaryocytes and few platelets present. The patient returned on October 6 for follow-up, with CBC showing a WBC of 6.27 × 10^9^/L, HGB of 92 g/L, and PLT of 13 × 10^9^/L, without hematuria, hematemesis, or melena. Immediate treatment with interleukin-11 injection, recombinant human thrombopoietin injection, and platelet transfusion was initiated sequentially. CBC reassessment on October 9 showed a WBC of 2.3 × 10^9^/L, neutrophils of 0.76 × 10^9^/L, HGB of 91 g/L, and PLT of 8.0 × 10^9^/L. Small subcutaneous bleeding spots were observed in the lower extremities, prompting the addition of oral pirarubicin at a dosage of 5mg per day to prevent bleeding. The patient’s bone marrow suppression persisted, with temporary improvement following platelet transfusions but subsequent decrease. CBC on October 19 showed a WBC of 1.69 × 10^9^/L, neutrophils of 0.9 × 10^9^/L, HGB of 78 g/L, and PLT of 12.0 × 10^9^/L. The dosage of pirarubicin was adjusted to 7.5 mg per day, and treatment with leukocyte elevation agents and interleukin-11 injection was continued. Until November 28, the patient’s PLT remained persistently low, with incomplete recovery of WBC and HGB levels. No further chemotherapy was administered during this period.

And MDS were subsequently confirmed by diagnosis of the bone marrow biopsy in Hangzhou First People’s Hospital. Consequently, patient simultaneously began therapy with a twice-daily regimen of 20 mg oral tretinoin tablets, a ter-in-die regimen of 5 mg oral folic acid tablets, a quaque-die regimen of 20 mg oral avatrombopag tablets and a twice-weekly regimen of recombinant human erythropoietin injection for subcutaneous injection, and a partial outcome was achieved. Thus far, no other complications have been recorded.

### 2.2. Investigations

This patient had a 4-year controllable hypertension history, a 40-year history of smoking and a 40-year drinking history. And he denied any history of exposure to dust, radioactive substances and poisons and denied any family history of malignant tumors. After admission, laboratory tests showed decreased white blood cell, hemoglobin and blood platelet. A bone marrow examination revealed multilineage dysplasia and 9.5% primitive blood cells. Cytogenetic analysis disclosed complex anomalies of chromosome.

### 2.3. Treatment

During the XELOX treatment period, the patient’s hematological function continued to deteriorate (Table 2). The diagnosis of MDS was made by bone marrow biopsy and treatment with tretinoin tablets, folic acid tablets, avatrombopag tablets, and recombinant human erythropoietin injection was initiated. Subsequently, significant hematological improvements were achieved.

**Table 2:** The results of hematological function analysis.

Date	White blood cell (×10^9^/L)	Hemoglobin (g/L)	Platelets (×10^9^/L)
February 24, 2021	4.64	132	399
March 10, 2021	3.18	128	259
March 15, 2021	2.5	113	178
March 22, 2021	4.98	126	236
March 26, 2021	3.1	117	209
April 3, 2021	13.6	114	148
April 10, 2021	9.36	108	34
April 17, 2021	2.66	101	146
April 19, 2021	5.68	104	220
May 6, 2021	2.93	121	149
May 11, 2021	4.57	109	22
July 23, 2021	2.87	111	152
July 30, 2021	2.99	107	64
August 12, 2021	2.81	93	69
September 14, 2021	2.76	103	11
September 15, 2021	2.34	91	65
September 18, 2021	0.36	88	37
September 22, 2021	2.63	92	21
September 25, 2021	2.08	91	14
September 30, 2021	2	90	54
October 6, 2021	6.27	92	13
October 9, 2021	2.3	91	8
October 19, 2021	1.69	78	12
November 19, 2021	0.98	76	40
November 27, 2021	2.18	79	68
December 20, 2021	3.4	102	16
February 5, 2022	3.13	101	7
March 1, 2022	2.08	79	43

## 3. Discussion

GC is the second most frequently diagnosed cancer and still a significant burden in China with an estimated 41,532 deaths in 2021, accounting for >35% of the worldwide annual incidence.^[2]^ And the early diagnosis of gastric cancer has not been carried out in a large scale in most of China, which leading to low diagnosis rate.^[3]^ However, in recent years, many great advances have been made in gastric cancer, particularly with respect to diagnosis, targeted therapy and treatment technology. Several Phase III randomized, controlled trials (RCTs) have already demonstrated significant survival advantages with chemotherapy in GC patients.^[4–6]^

According to the guidelines of Chinese Society of Clinical Oncology (CSCO), chemotherapy is the first-line therapy for approximately 80% of HER2-negative patients. The oral CAP combined with oxaliplatin treatment for gastric cancer showed a benefit for adjuvant chemotherapy after D2 gastrectomy first in 2012.^[7]^ The overall survival in this trial increased from 78% to 83% while three-year survival increased from 59% to 74% with adjuvant chemotherapy. In this case, 3 of the 19 lymph nodes on the lesser curvature of the stomach had metastasized. Noh SH had already conducted that combination chemotherapy with the XELOX regimen (capecitabine plus oxaliplatin) is more appropriate for node-positive disease, which is consistent with our treatment.^[8]^ In the CLASSIC study, XELOX chemotherapy also led to an obvious improvement in survival compared with surgery alone (5-year disease-free survival [DFS]: 67 vs 53%, *P* < .0001) and a 34% decline in the cancer-related death (*P* < .05).

The MDSs are clonal disorders with dysplasia of at least 1 myeloid cell line, indolent disease courses, peripheral blood cytopenia, and a propensity to eventually progress into acute myeloid leukemia.^[9,10]^ The erythropoiesis stimulating agents, including recombinant humanized erythropoietin, are used to improve anemia state in 15% to 40% of patients with lower-risk MDS.^[11]^ There are a series of cases reporting that MDS occurs in combination with solid tumors. Paula et al reported 2 cases associated with synchronous gastric cancers in a young and an elderly patient.^[12]^ Meng D et al reported a 47-year-old woman suffering from cervical carcinoma and MDS.^[13]^ Eun Joo Lim et al reported an endoscopic submucosal dissection (ESD) case for early gastric cancer in the patient with myelodysplastic syndrome.^[14]^ However, it is extremely rare that myelodysplastic syndrome develops in a gastric cancer patient.

Capecitabine and gemcitabine have been the mainstream therapeutic drugs for advanced colon, gastric and breast cancer. While the patients treated with capecitabine had already experienced some adverse drug reactions at some point during the treatment course, including gastrointestinal toxicity, hand-foot syndrome, diarrhea, hypertriglyceridemia, nausea, and/or vomiting.^[15]^ And adjuvant XELOX chemotherapy has been widely applied in GC patients to lower the incidence and severity of adverse events of capecitabine.^[16]^ However, Grade III or IV adverse events in the surgery following capecitabine group were observed mainly in the metastatic breast cancer, including lymphopenia, neutropenia, and thrombocytopenia.^[17]^ A prospective clinical study of gastroenteropancreatic neuroendocrine tumor patients treated with 177Lu-octreotate peptide receptor radionuclide therapy in combination with capecitabine and temozolomide demonstrated that acute toxicity most often affects platelets during the first cycle of treatment.^[18]^

In this case, acute MDS occurred during the early XELOX courses, which was partially relieved by corresponding drug treatment. The mechanism of MDS caused by XELOX could be impaired renal function and baseline cytopenias, which frequently appearing in the course of the elderly. Although the incidences of secondary MDS is low, they could significantly reduce quality of life, and as such, should be paid attention. To the best of our knowledge, although GC patients with neutropenia or leucopenia have been previously reported, few cases of GC patients with MDS after the end of XELOX treatment have been reported. Our article presents an additional case in a Chinese man and this seems to be relatively uncommon. However, many questions remain to be answered. For example, is there a relationship between GC and MDS? How to make a definite diagnosis for MDS in the GC patients? The presence of symptoms which are associated with MDS, such as significant peripheral blood cytopenia and neutropenia should raise clinical suspicion, leading to an extensive medical checkup. It is vital to point out the probabilities of co-existence of other neoplasms with MDS.

## Author contributions

**Conceptualization:** Hang-ping Chen, Guo-fei Ren.

**Methodology:** Yi-fan Qian, Hang-ping Chen.

**Supervision:** Guo-fei Ren.

**Writing – original draft:** Yi-fan Qian, Hang-ping Chen.

**Writing – review & editing:** Yi-fan Qian, Guo-fei Ren.
